# P48 Service evaluation of disease burden of, and damage caused by, wrist arthritis in a single centre paediatric cohort of JIA

**DOI:** 10.1093/rap/rkac067.048

**Published:** 2022-09-28

**Authors:** Eman Al Masroori, Eslam Al-Abadi

**Affiliations:** Birmingham Women’s and Children’s Hospital NHS Foundation Trust, Birmingham, United Kingdom; Birmingham Women’s and Children’s Hospital NHS Foundation Trust, Birmingham, United Kingdom

## Abstract

**Introduction/Background:**

Wrist synovitis is associated with more severe disease and less drug-free remission periods, therefore, the objective of this study was to investigate the demographic, disease course, and complexity of wrist disease in children being treated for Juvenile Idiopathic Arthritis (JIA).

**Description/Method:**

A single-centre, retrospective case note review of all children and adolescents diagnosed with JIA, who received steroid joint injections of their wrists from 2014 to 2019. All patients received standard therapy and underwent regular follow-up at Birmingham Children’s Hospital. Inclusion criteria were those with an established diagnosis of any JIA-subtype according to the International League of Associations for Rheumatology (ILAR) classification. Patients were excluded if (i) the data were incomplete, (ii) they were over the age of 16y at diagnosis, or (iii) the eventual diagnosis was not JIA. Clinical information was gathered from case notes, departmental databases and hospital clinical systems on to a standardised proforma. Information collected were demographics, JIA subtype, immunology, age at diagnosis, disease duration, number of wrist injections and outcome of every wrist MRI performed. All treatments including intraarticular injections were administered by a paediatric Rheumatologist while MRIs were reviewed by an experienced senior Radiologist.

**Discussion/Results:**

Of all 94 eligible patients, 88 (94%) were included in this study; 71 girls, with a mean age at diagnosis of 6 years (0-15 years). Wrist arthritis was more common in seronegative polyarthritis (40%) and extended oligoarthritis (23%) but least pronounced in Enthesitis-related arthritis (2%). 85 (95.6%) received Methotrexate therapy with a mean of 10 months from time of diagnosis. 77 patients (88%) required a step-up from MTX, with Etanercept (88%) being the most popular option as a first-line biologics, followed by Adalimumab (11%). All patients who had Adalimumab as a first biologics had evidence of uveitis.

Wrist disease was present at diagnosis in 60/88 (68%) of our cohort. 66 patients (75%) had bilateral wrist disease. Over one-quarter of patients who developed wrist arthritis within the first year of diagnosis were young children (0-2 years). A second predominate group was adolescent (10-15 years) accounting for 33% of our cohort. The mean (IQR) duration between diagnosis and first clinical evidence of wrist synovitis was 15 months (1–17). Patients had a median of 3 years from diagnosis until development of first radiological evidence of erosive changes.

12 children (14%) from this cohort underwent a laparoscopic synovectomy for their wrist damage between 2017 and 2021, out of which 9 had polyarticular involvement while two had extended and one had persistent oligoarticular arthritis. Half of the patients reported improvement in pain and Health Assessment Questionnaire (HAQ) scores after synovectomy, while three remained stable at 0 score, and 3 had insufficient data.

**Key learning points/Conclusion:**

Treatment of wrist arthritis can be challenging to both professionals and health care systems, emphasizing the need for clear steps to improve early recognition and treatment. Wrist disease at presentation was relatively common in our cohort and damage once occurred was irreversible with patients continuing to have ongoing disease activity despite optimization of their treatment. Furthermore, MRI as shown in our analysis, can accurately detect early erosive changes and inform management decision.

